# Supplementary material for: IL-17B protects against uropathogenic E. coli-induced kidney injury via macrophage infiltration modulation
Source: Microbiol Spectr. 2026 May 1;14(6):e02244-25. doi: 10.1128/spectrum.02244-25 (PMC13228029; doi:10.1128/spectrum.02244-25)
Supplement: Supplemental materials — Fig. S1 to S4, Tables S1 and S2, and the list of primers and reagents. [file spectrum.02244-25-s0001.pdf]

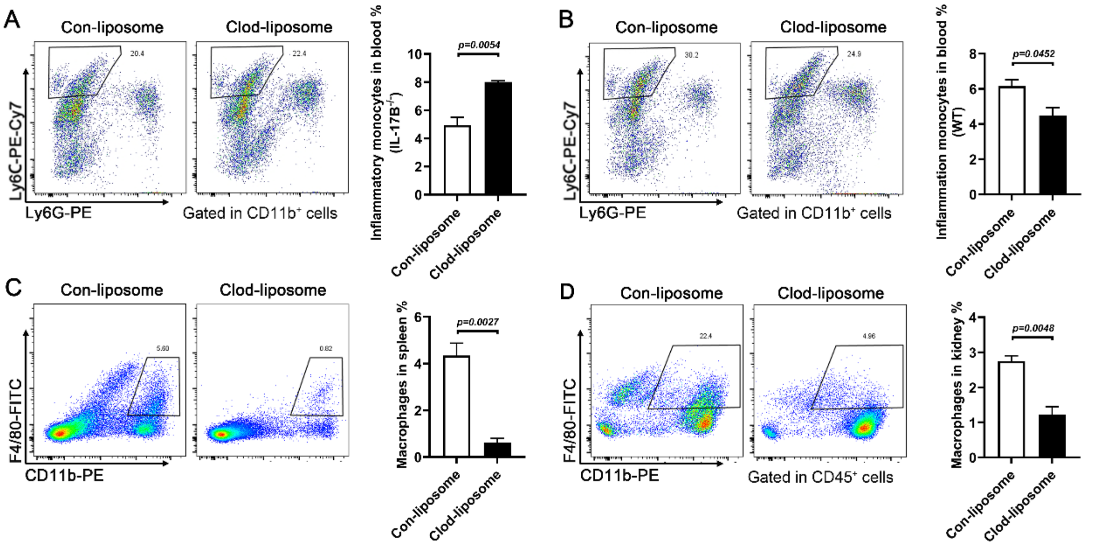

Supplement 1. Splenic macrophages and renal macrophages were depleted by Clodronate liposome

IL-17B$^{-/-}$ and WT mice were treated intravenously with Clod-liposomes or Con-liposomes and then infected with CFT073. Flow cytometry was used to assess the depletion efficiency (n = 3). (A-B) The percentages of inflammatory monocytes in the blood of IL-17B$^{-/-}$ and WT mice. (C-D) The percentages of macrophages in the kidney and spleen of IL-17B$^{-/-}$ mice. Data are presented as mean ± SEM. Statistical comparisons (A-D) were performed using a *t*-test.

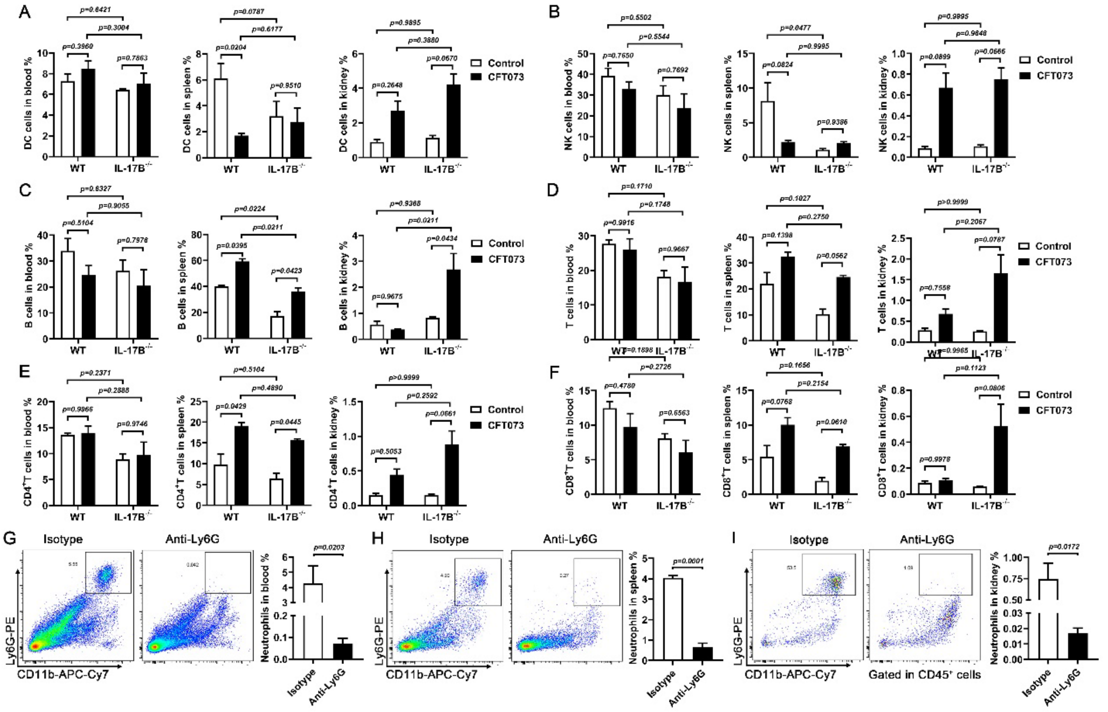

Supplement 2. The effect of IL-17B on various immune cells in the kidney during infection

(A-F) Proportions of DC cells, NK cells, B cells, and T cells in the peripheral blood, spleen, and kidneys of mice after infection with 1 × 10^8 CFU of CFT073. (G-I) IL-17B$^{-/-}$ mice were treated intravenously with anti-Ly6G antibody or isotype antibody and then infected with CFT073. Flow

cytometry was used to assess the depletion efficiency (n = 3). Data are presented as mean ± SEM. Statistical comparisons (A-F) were performed using two-way ANOVA. Data are presented as mean ± SEM. Statistical comparisons (A-F) were conducted using a two-way ANOVA. (G-I) were analyzed using a *t*-test.

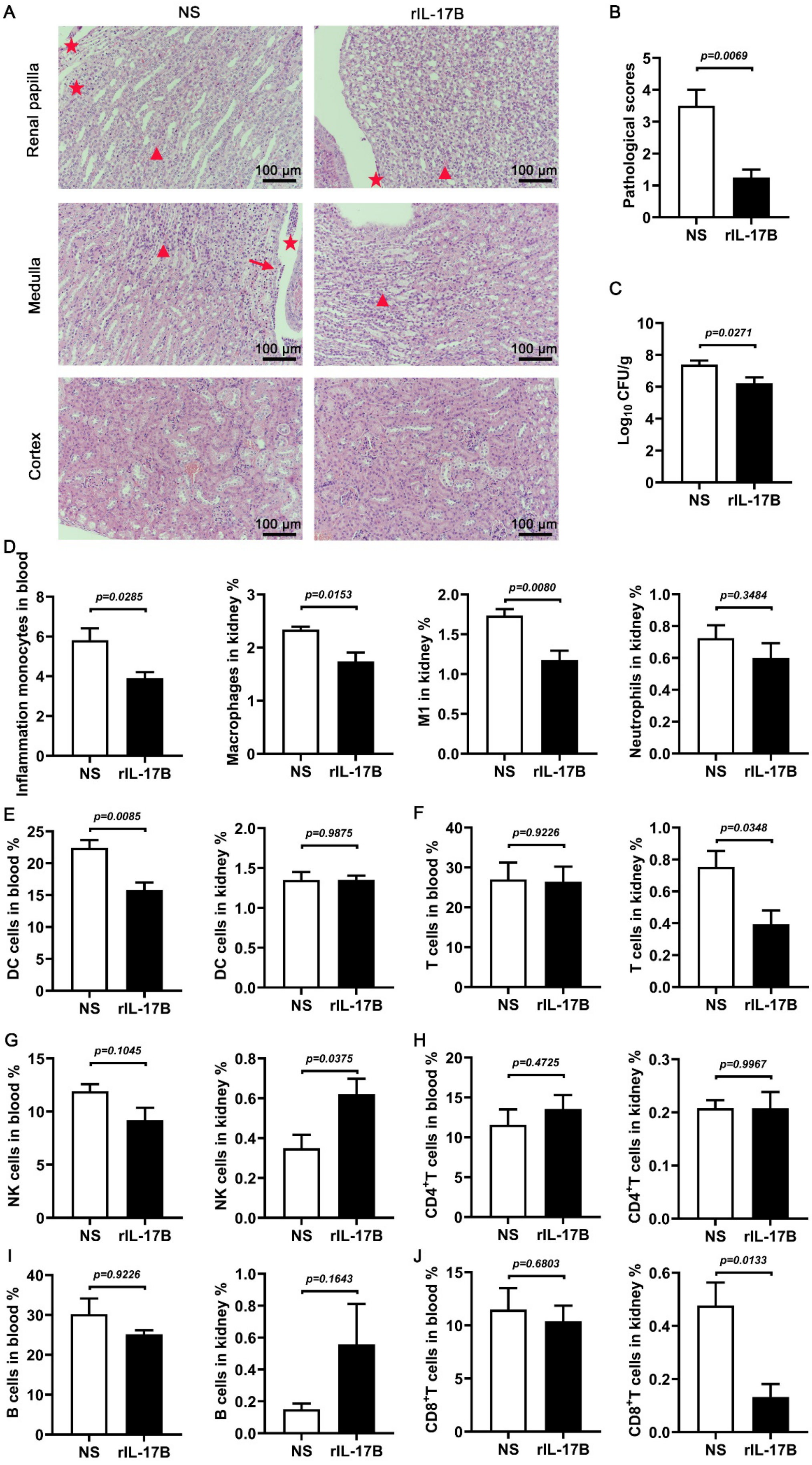

A
NS
rIL-17B
Renal papilla
Medulla
Cortex
100 μm
B
Pathological scores
p=0.0069
NS
rIL-17B
C
Log10 CFU/g
p=0.0271
D
Inflammation monocytes in blood
p=0.0285
Macrophages in kidney %
p=0.0153
M1 in kidney %
p=0.0080
Neutrophils in kidney %
p=0.3484
E
DC cells in blood %
p=0.0085
DC cells in kidney %
p=0.9875
F
T cells in blood %
p=0.9226
T cells in kidney %
p=0.0348
G
NK cells in blood %
p=0.1045
NK cells in kidney %
p=0.0375
H
CD4+T cells in blood %
p=0.4725
CD4+T cells in kidney %
p=0.9967
I
B cells in blood %
p=0.9226
B cells in kidney %
p=0.1643
J
CD8+T cells in blood %
p=0.6803
CD8+T cells in kidney %
p=0.0133

Supplement 3. The effect of IL-17B on various immune cells in the kidney of WT mice during infection

WT mice were treated with rIL-17B and infected with 1 × 10^8 CFU of CFT073, administered twice at 3-hour intervals. (A-B) Representative H&E-stained kidney sections and histopathological injury scores. Triangles denote hemorrhage, arrows indicate tissue damage, and stars mark inflammatory cell infiltration. 200×, 100 μm (n=5). (C) Bacterial colonization in the kidney or bladder at 24 hours after infection (n=5). (D) Percentages of inflammatory monocytes in peripheral blood, and macrophages, neutrophils in the kidneys (n=5). (E-J) Proportions of DC cells, NK cells, B cells, and T cells in the peripheral blood, spleen, and kidneys of mice treated with rIL-17B. Data are presented as mean ± SEM. Statistical comparisons were conducted using a *t*-test (B-J).

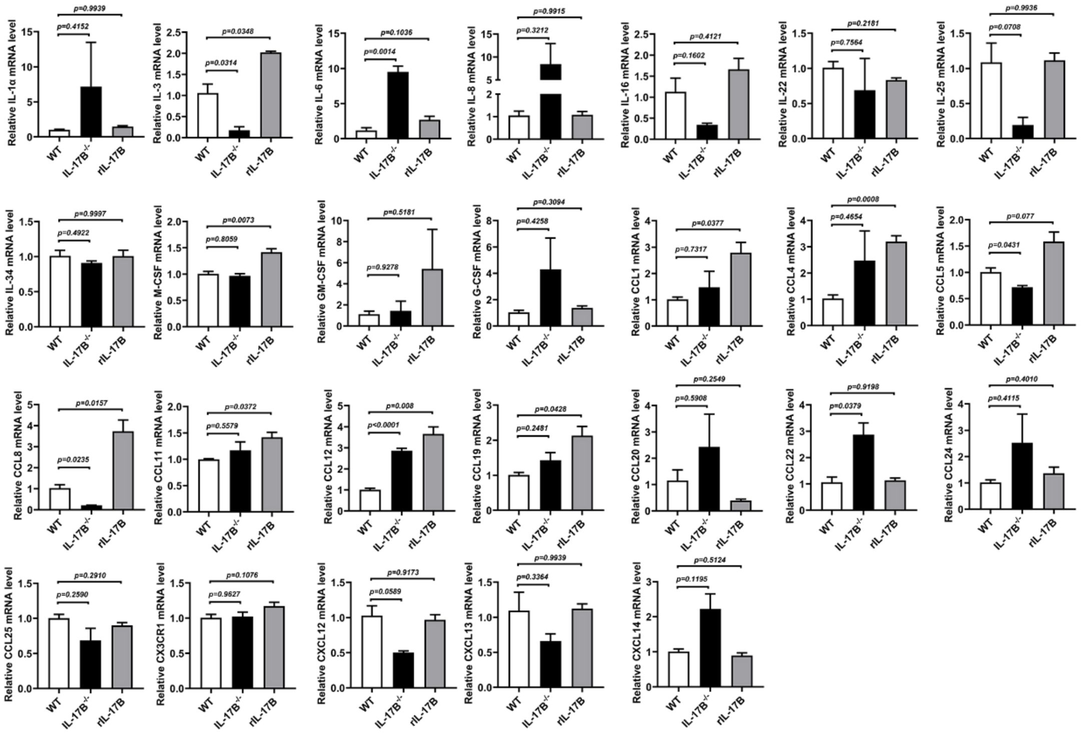

Supplement 4. IL-17B affected various chemokines in the kidney during CFT073 infection

WT and IL-$17B^{-/-}$ mice were infected with CFT073, and WT mice were treated with rIL-17B and CFT073. qPCR was used to detect the expression of various chemokines (n=3). Data are shown as mean ± SEM and analyzed by one-way ANOVA.

Supplementary Table 1. Primers used in this study

Primer	Sequence (5'-3')	Description
mIL-17B-FP	GAGTAAAGCCCTACGCTCGAA	For mouse IL-17B qRT-PCR
mIL-17B-RP	CTCCTCTTGTTGGACAACCAC	
mIL-17RB-FP	GGCTGCCTAAACCACGTAATG	For mouse IL-17RB qRT-PCR
mIL-17RB-RP	CCCGTTGAATGAGAATCGTGT	
mIL-25-FP	GGACAGGGACTTGAATCGGG	For mouse IL-25 qRT-PCR
mIL-25-RP	GAAGACCGTCTGGTTGTGGT	
mIL-17A-FP	CAGACTACCTCAACCGTTCCACT	For mouse IL-17A qRT-PCR
mIL-17A-RP	GCACTTCTCAGGCTCCCTCTTC	
mIL-17C-FP	TGACAGCCTATCCCAGAGGG	For mouse IL-17C qRT-PCR
mIL-17C-RP	ACCAGCCTAGAAGCAGGAGA	
mIL-17D-FP	AGCACACCCGTCTTCTCTC	For mouse IL-17D qRT-PCR
mIL-17D-RP	GCTGGAGTTCGCACTGTCC	
mIL-17F-FP	TGCTACTGTTGATGTTGGGAC	For mouse IL-17F qRT-PCR
mIL-17F-RP	AATGCCCTGGTTTTGGTTGAA	
mIL-17RA-FP	CAAGTTTCACTGGTGCTGCC	For mouse IL-17RA qRT-PCR
mIL-17RA-RP	TAGTCTGCAACTGGCTTGGG	
mIL-17RC-FP	TCTTGGGGCTGAGGTACAGA	For mouse IL-17RC qRT-PCR
mIL-17RC-RP	CGGACTTCAAGACCCCTGC	
mIL-17RD-FP	ACAGCAACACCACCAGGAAA	For mouse IL-17RD qRT-PCR
mIL-17RD-RP	GCACATCACAGTGAACAGGG	
mIL-17RE-FP	TGTGCTTCTCACAGCTCCAG	For mouse IL-17RE qRT-PCR
mIL-17RE-RP	ACGGTAGGCCAGGAGACAG	
mIL-1β-FP	GCAACTGTTCCTGAACTCAACT	For mouse IL-1β qRT-PCR
mIL-1β-RP	ATCTTTTGGGGTCCGTCAACT	
mIL-6-FP	TAGTCCTTCCTACCCCAATTTCC	For mouse IL-6 qRT-PCR
mIL-6-RP	TTGGTCCTTAGCCACTCCTTC	
mIL-12/23p40-FP	TGGTTTGCCATCGTTTTGCTG	For mouse IL-12/23p40 qRT-PCR
m IL-12/23p40-RP	ACAGGTGAGGTTCACTGTTTCT	
mTNFα-FP	CCCTCACACTCAGATCATCTTCT	For mouse TNFα qRT-PCR
mTNFα-RP	GCTACGACGTGGGCTACAG	
miNOS-FP	GTTCTCAGCCCAACAATACAAGA	For mouse iNOS qRT-PCR
miNOS-RP	GTGGACGGGTCGATGTCAC	
mIFNγ-FP	ATGAACGCTACACACTGCATC	For mouse IFN-γ qRT-PCR
mIFNγ-RP	CCATCCTTTTGCCAGTTCCTC	
mIL-10-FP	GCTCTTACTGACTGGCATGAG	For mouse IL-10 qRT-PCR
mIL-10-RP	CGCAGCTCTAGGAGCATGTG	
mTGF-β-FP	CTCCCGTGGCTTCTAGTGC	For mouse TGF-β qRT-PCR
mTGF-β-RP	GCCTTAGTTTGGACAGGATCTG	
mIL-1α-FP	CGAAGACTACAGTTCTGCCATT	For mouse IL-1α qRT-PCR
mIL-1α-RP	GACGTTTCAGAGGTTCTCAGAG	
mIL-3-FP	GCCTGCCTACATCTGCGAAT	For mouse IL-3 qRT-PCR
mIL-3-RP	TCAGTTTCTTCCGAAAGTCATCC	

mIL-8-FP	TGGGTGAAGGCTACTGTTGG	For mouse IL-8 qRT-PCR
mIL-8-RP	GTCTCCCGAATTGGAAAGGGA	
mIL-16-FP	AAGAGCCGGAAATCCACGAAA	For mouse IL-16 qRT-PCR
mIL-16-RP	GTCTCAAAAGGGTCAGGGTACT	
mIL-22-FP	ATGAGTTTTTCCCTTATGGGGAC	For mouse IL-22 qRT-PCR
mIL-22-RP	GCTGGAAGTTGGACACCTCAA	
mIL-34-FP	TTGCTGTAAACAAAGCCCCAT	For mouse IL-34 qRT-PCR
mIL-34-RP	CCGAGACAAAGGGTACACATTT	
mM-CSF-FP	GTGTCAGAACACTGTAGCCAC	For mouse M-CSF qRT-PCR
mM-CSF-RP	TCAAAGGCAATCTGGCATGAAG	
mG-CSF-FP	ATGGCTCAACTTTCTGCCCAG	For mouse G-CSF qRT-PCR
mG-CSF-RP	CTGACAGTGACCAGGGGAAC	
mGM-CSF-FP	GGCCTTGGAAGCATGTAGAGG	For mouse GM-CSF qRT-PCR
mGM-CSF-RP	GGAGAACTCGTTAGAGACGACTT	
mCCL1-FP	GGCTGCCGTGTGGATACAG	For mouse CCL1 qRT-PCR
mCCL1-RP	AGGTGATTTTGAACCCACGTTT	
mCCL2-FP	TTAAAAACCTGGATCGGAACCAA	For mouse CCL2 qRT-PCR
mCCL2-RP	GCATTAGCTTCAGATTTACGGGT	
mCCL3-FP	TTCTCTGTACCATGACACTCTGC	For mouse CCL3 qRT-PCR
mCCL3-RP	CGTGGAATCTTCCGGCTGTAG	
mCCL4-FP	CAGCCCTGATGCTTCTCACT	For mouse CCL4 qRT-PCR
mCCL4-RP	GGGAGACACGCGTCCTATAAC	
mCCL5-FP	GCTGCTTTGCCTACCTCTCC	For mouse CCL5 qRT-PCR
mCCL5-RP	TCGAGTGACAAACACGACTGC	
mCCL7-FP	GCTGCTTTCAGCATCCAAGTG	For mouse CCL7 qRT-PCR
mCCL7-RP	CCAGGGACACCGACTACTG	
mCCL8-FP	TCTACGCAGTGCTTCTTTGCC	For mouse CCL8 qRT-PCR
mCCL8-RP	AAGGGGGATCTTCAGCTTTAGTA	
mCCL11-FP	GAATCACCAACAACAGATGCAC	For mouse CCL11 qRT-PCR
mCCL11-RP	ATCCTGGACCCACTTCTTCTT	
mCCL12-FP	ATTTCCACACTTCTATGCCTCCT	For mouse CCL12 qRT-PCR
mCCL12-RP	ATCCAGTATGGTCCTGAAGATCA	
mCCL19-FP	GGGGTGCTAATGATGCGGAA	For mouse CCL19 qRT-PCR
mCCL19-RP	CCTTAGTGTGGTGAACACAACA	
mCCL20-FP	ACTGTTGCCTCTCGTACATACA	For mouse CCL20 qRT-PCR
mCCL20-RP	GAGGAGGTTCACAGCCCTTTT	
mCCL22-FP	CTCTGCCATCACGTTTAGTGAA	For mouse CCL22 qRT-PCR
mCCL22-RP	GACGGTTATCAAAACAACGCC	
mCCL24-FP	ATTCTGTGACCATCCCCTCAT	For mouse CCL24 qRT-PCR
mCCL24-RP	TGTATGTGCCTCTGAACCCAC	
mCCL25-FP	TTACCAGCACAGGATCAAATGG	For mouse CCL25 qRT-PCR
mCCL25-RP	CGGAAGTAGAATCTCACAGCAC	
mCXCL12-FP	TGCATCAGTGACGGTAAACCA	For mouse CXCL12 qRT-PCR
mCXCL12-RP	TTCTTCAGCCGTGCAACAATC	

mCXCL13-FP	GGCCACGGTATTCTGGAAGC	For mouse CXCL13 qRT-PCR
mCXCL13-RP	GGGCGTAACTTGAATCCGATCTA	
mCXCL14-FP	GAAGATGGTTATCGTCACCACC	For mouse CXCL14 qRT-PCR
mCXCL14-RP	CGTTCCAGGCATTGTACCACT	
mCX3CL-FP	CTGCCCTCACTAAAAATGGTGG	For mouse CX3CL qRT-PCR
mCX3CL-RP	AATGTGGCGGATTCAGGCTT	
mCCR2-FP	ATCCACGGCATACTATCAACATC	For mouse CCR2 qRT-PCR
mCCR2-RP	CAAGGCTCACCATCATCGTAG	
mCCR3-FP	TCAACTTGGCAATTTCTGACCT	For mouse CCR3 qRT-PCR
mCCR3-RP	CAGCATGGACGATAGCCAGG	
mCCR7-FP	TGTACGAGTCGGTGTGCTTC	For mouse CCR7 qRT-PCR
mCCR7-RP	GGTAGGTATCCGTCATGGTCTTG	
mβ-actin-FP	GGCTGTATTCCCCTCCATCG	For mouse β-actin qRT-PCR
mβ-actin-RP	CCAGTTGGTAACAATGCCATGT	

Supplementary Table 2. The antibodies used in this study for flow cytometry

**Name**	Clone, Item Number	**Manufacturer**
CD16/32	93, 14-0161-82	eBioscience
CD11b-FITC	M1/70, 101206	BioLegend
CD11b-PE	M1/70, 101208	BioLegend
F4/80-PE	BM8, 123110	BioLegend
F4/80-FITC	BM8, 123108	BioLegend
CD86-APC/Cy7	GL-1, 105030	BioLegend
CD206-APC	C068C2, 141708	BioLegend
Ly6C-APC	HK1.4, 128016	BioLegend
Ly6C-PE-Cy7	HK1.4, 128018	BioLegend
Ly6G-PE	1A8, 127608	BioLegend
CD11c-APC	N418, 117310	BioLegend
B220-FITC	RA3-6B2, 103206	BioLegend
NK1.1-PE	PK136, 108708	BioLegend
CD3-PE	17A2, 100206	BioLegend
CD4-FITC	GK1.5, 100406	BioLegend
CD8-APC	53-6.7, 100712	BioLegend
CD45-BV421	30-F11, 103134	BioLegend
